# Role of methanotrophic communities in atmospheric methane oxidation in paddy soils

**DOI:** 10.3389/fmicb.2024.1481044

**Published:** 2024-11-06

**Authors:** Yan Zheng, Yuanfeng Cai, Zhongjun Jia

**Affiliations:** ^1^College of Food and Bioengineering, Zhengzhou University of Light Industry, Zhengzhou, Henan, China; ^2^State Key Laboratory of Soil and Sustainable Agriculture, Institute of Soil Science, Chinese Academy of Sciences, Nanjing, Jiangsu, China

**Keywords:** atmospheric methane oxidation, high-affinity methanotrophs, methanotrophic biogeography, paddy soils, stable isotope probing

## Abstract

Wetland systems are known methane (CH_4_) sources. However, flooded rice fields are periodically drained. The paddy soils can absorb atmospheric CH_4_ during the dry seasons due to high-affinity methane-oxidizing bacteria (methanotroph). Atmospheric CH_4_ uptake can be induced during the low-affinity oxidation of high-concentration CH_4_ in paddy soils. Multiple interacting factors control atmospheric CH_4_ uptake in soil ecosystems. Broader biogeographical data are required to refine our understanding of the biotic and abiotic factors related to atmospheric CH_4_ uptake in paddy soils. Thus, here, we aimed to assess the high-affinity CH_4_ oxidation activity and explored the community composition of active atmospheric methanotrophs in nine geographically distinct Chinese paddy soils. Our findings demonstrated that high-affinity oxidation of 1.86 parts per million by volume (ppmv) CH_4_ was quickly induced after 10,000 ppmv high-concentration CH_4_ consumption by conventional methanotrophs. The ratios of 16S rRNA to rRNA genes (rDNA) for type II methanotrophs were higher than those for type I methanotrophs in all acid-neutral soils (excluding the alkaline soil) with high-affinity CH_4_ oxidation activity. Both the 16S rRNA:rDNA ratios of type II methanotrophs and the abundance of ^13^C-labeled type II methanotrophs positively correlated with high-affinity CH_4_ oxidation activity. Soil abiotic factors can regulate methanotrophic community composition and atmospheric CH_4_ uptake in paddy soils. High-affinity methane oxidation activity, as well as the abundance of type II methanotroph, negatively correlated with soil pH, while they positively correlated with soil nutrient availability (soil organic carbon, total nitrogen, and ammonium-nitrogen). Our results indicate the importance of type II methanotrophs and abiotic factors in atmospheric CH_4_ uptake in paddy soils. Our findings offer a broader biogeographical perspective on atmospheric CH_4_ uptake in paddy soils. This provides evidence that periodically drained paddy fields can serve as the dry-season CH_4_ sink. This study is anticipated to help in determining and devising greenhouse gas mitigation strategies through effective farm management in paddy fields.

## 1 Introduction

The atmospheric concentration of methane (CH_4_), a significant greenhouse gas, has increased from 0.82 parts per million volume (ppmv) in 1841 to over 1.86 ppmv in 2019 (Etheridge et al., 1992; IPCC, 2021). Wetlands are known CH_4_ sources with high global warming potential (IPCC, 2021). However, flooded paddy soils (Conrad, 2007), Arctic wetlands (Voigt et al., 2023), and mire-wetlands (Wang et al., 2023), known as CH_4_ emitters, act as CH_4_ sinks during dry periods. Multiple interacting factors control the atmospheric CH_4_ concentrations in wetlands (Maucieri et al., 2017). Hence, an investigation of potential soil factors that increase wetland soil CH_4_ sinks is needed to mitigate the greenhouse effect.

Water content plays a critical role in controlling CH_4_ emissions in wetlands by regulating the relative proportions of anaerobic zones for CH_4_ production and aerobic zones for CH_4_ oxidation within the soil column (Schultz et al., 2023). Soil drying promotes atmospheric CH_4_ uptake in Arctic soils (Voigt et al., 2023). Higher CH_4_ uptake is linked to increased availability of soil nutrients (Lee et al., 2023; Voigt et al., 2023). The soil CH_4_ sink increases with enhanced soil nitrogen (Voigt et al., 2023). Nitrogen promotes CH_4_ oxidation by stimulating the growth of methane-oxidizing bacteria (methanotroph) in paddy soils (Zheng et al., 2014; Nijman et al., 2021). Soil organic carbon (SOC) decomposition can provide an alternative carbon substrate that promotes the growth of methanotrophs, thereby mediating the atmospheric CH_4_ uptake (Lee et al., 2023). SOC decomposition responds to various complex factors in soil ecosystems (Lin et al., 2023). Increased temperatures can stimulate SOC decomposition (Zhao et al., 2023; Nazir et al., 2024). Temperature positively affects soil CH_4_ sink (Lee et al., 2023). Soil pH may also influence the CH_4_ oxidation activity by regulating the methanotrophic community (Shiau et al., 2018; Zhao et al., 2020).

Methane-oxidizing bacteria-based microbial CH_4_ oxidation is the sole known biological sink of atmospheric CH_4_ (Saunois et al., 2020). Almost all cultivated aerobic methanotrophs belong to *Proteobacteria* and are divided into two major subgroups: type I methanotrophs (*Gammaproteobacteria*) and type II methanotrophs (*Alphaproteobacteria*) (Dedysh and Knief, 2018). Established type II methanotrophs, such as *Methylocystis*, *Methylosinus*, and *Methylocapsa* (Baani and Liesack, 2008; Tveit et al., 2019; Tikhonova et al., 2021) and certain conventional type I methanotrophs can oxidize atmospheric CH_4_ (Benstead et al., 1998). Type I and type II methanotrophs predominate under different environmental conditions, owing to their distinct life strategies (Ho et al., 2013). Type I methanotrophs are generally favored by high nutrient availability (Steenbergh et al., 2010; Zheng et al., 2014), whereas type II methanotrophs are more competitive under oligotrophic conditions (Cai et al., 2016). Abiotic factors affect soil atmospheric CH_4_ levels by regulating the methanotrophic community (Conrad, 2007; Lee et al., 2023; Voigt et al., 2023). Therefore, understanding the roles of different methanotrophic taxa in atmospheric CH_4_ uptake will provide deeper insights into the mitigation capacity of methanotrophic communities.

Rice fields play a central role in determining the global CH_4_ budget (IPCC, 2021). The varying water content in the rice fields results in notable fluctuations in soil CH_4_ concentrations (Conrad, 2007). A nature wetland conversation to the upland can turn a CH_4_ source into a CH_4_ sink (Wang et al., 2023). The rice fields are similar to aerated upland soils during the dry seasons (Conrad, 2007). The paddy soil CH_4_ source under flooded conditions turns into an atmospheric CH_4_ sink during the dry seasons (Singh et al., 1996). Paddy soils oxidize atmospheric CH_4_ only after incubation under conditions involving high CH_4_ (Yan and Cai, 1997). Conventional methanotrophs in a typical paddy soil can rapidly induce high-affinity CH_4_ oxidation (1.86 ppmv) when exposed to high CH_4_ concentrations (Cai et al., 2016). The aforementioned previous studies provide evidence that periodically drained paddy fields can act as an atmospheric CH_4_ sink. Therefore, the investigation of broader biogeographical data is needed to improve our understanding of the factors related to atmospheric CH_4_ uptake in paddy soils. This study will help determine effective farm management strategies to enhance atmospheric CH_4_ sink in paddy fields, providing a climate mitigation strategy.

Here, we aimed to assess high-affinity CH_4_ oxidation activity and explore the community composition of active atmospheric methanotrophs. To this end, we selected nine paddy soils across three climate zones from the primary rice production areas in China. The potential activity of the methanotrophic community in the paddy soils was explored using the 16S rRNA:rDNA ratios of methanotrophs based on high-throughput sequencing and documenting their ^13^CH_4_-labeled relative abundance complemented by DNA- and RNA-based stable isotope probing (SIP). The 16S rRNA:rDNA ratios may offer deeper insights into bacterial community activity than those of abundance alone (Campbell and Kirchman, 2013). In combination with high-throughput sequencing, DNA- and RNA-based SIP can target active methanotrophic communities by providing the growth substrate ^13^CH_4_ (Dumont et al., 2011). To our best knowledge, this is the first large-scale study to analyze the potential atmospheric CH_4_ uptake activity and ecological roles of different methanotrophs in atmospheric methane oxidation in paddy soils.

## 2 Materials and methods

### 2.1 Sampling sites

**FIGURE 1:**
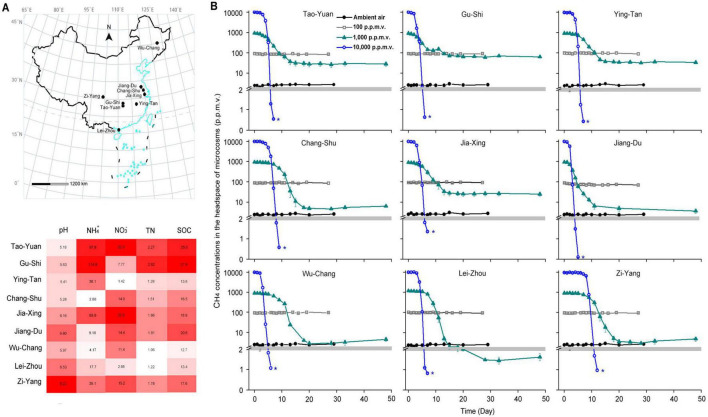
Emergence of high-affinity methane (CH_4_) oxidation in paddy soils sampled from China. **(A)** The sampling locations and soil abiotic factors of paddy soils in China. **(B)** CH_4_ consumption of paddy soils during incubation with various initial CH_4_ concentrations in the headspace of microcosms. The blue asterisks indicate the soil samples used to verify high-affinity CH_4_ oxidation activity in Figure 2 and to explore methanotrophic activity in Figure 3. The error bars represent standard deviations of triplicate microcosms. NO_3_^–^, nitrate (μg N g^–1^ dry weight soil [*d.w.s.*]); NH_4_^+^, ammonium (μg N g^–1^ *d.w.s.*); TN, total nitrogen (mg g^–1^ *d.w.s.*); and SOC, soil organic carbon (mg g^–1^ *d.w.s.*).

Paddy soils were sampled from nine different sites across the primary rice production areas in China (Figure 1A). The annual mean temperatures (AMT) ranged from 3.0 to 24.1°C at the sampling sites (Supplementary Table 1). All the sites have been used for rice cultivation for over 50 years. Soils were collected from each site immediately after the rice harvest, when the paddy fields had been drained. Five soil blocks, 20 meters apart from each other, were collected, and mixed to obtain a composite soil sample from each site. For each soil block, bulk soil from a depth of 0–15 cm was collected using a stainless-steel corer with an inner diameter of 7 cm. The composite soils were transported on ice to the laboratory and stored at 4°C for the analysis of soil environmental factors and incubation experiments. The soils were passed through a 2-mm sieve prior to incubation.

### 2.2 Soil variables

Soil pH was measured in a 1:2.5 (w/v) soil-to-water suspension using a Mettler Toledo 320-S pH meter (Mettler Toledo Instruments, Shanghai, China). Soil inorganic nitrogen, extracted using 2 mol/L KCl, was determined using a Skalar San Plus segmented flow analyser (Skalar, Breda, Netherlands). Soil total nitrogen (TN) and SOC were measured using a Vario Max CN element analyser (Elementar, Langenselbold, Germany). The total quantity of water absorbed by the soil is estimated using the water-holding capacity, which can be measured by the soil-cutting ring method (Yang et al., 2023). Briefly, the soil samples were oven-dried at 105°C for 8 h and then placed into a container. The dried soils were treated with water absorption. After soaking in water for 24 h, the weights of soaked soil samples were measured. The soaked soils were oven-dried at 105°C for 8 h until a constant weight was recorded. The weights of dry soil were recorded to determine the water content, which was then used to calculate the maximum water-holding capacity. The maximum water-holding capacity for each paddy soil is described in Supplementary Table 1.

### 2.3 Microcosm construction

The soil moisture of each 300 g of soil sample was adjusted to 60% maximum water-holding capacity and preincubated in an incubator for four days under ambient air conditions at 28°C in the dark. The incubator temperature (28°C) was monitored throughout the preincubation period. The fluctuation of temperature was within ± 0.1°C. Preincubated paddy soils were used as the initial soils (day 0). Before the development of a microcosm, water loss was replenished to maintain 60% maximum water-holding capacity in each soil.

The microcosms were constructed by adding 6.0 g (dry weight) of preincubated soil to a serum bottle (120 mL) capped with a gas-tight butyl rubber stopper. Identical microcosms were created using initial CH_4_ concentrations of 10,000, 1,000, 100, and 2 ppmv (ambient air) to mimic the fluctuating CH_4_ concentrations in periodically drained rice fields. The microcosms were placed into the incubator. The treatments were conducted at 60% maximum water holding capacity and 28°C in the dark throughout the incubation. Water loss could be generated as tiny droplets of water on the inner walls of the microcosm bottles during incubation. To maintain the soil moisture, we gently shook the bottles 3–5 times using our hands to return the water to the soil.

For the 10,000 ppmv CH_4_-amended soils, the ^12^CH_4_ (control) and ^13^CH_4_ SIP treatments were incubated with ^12^CH_4_ and ^13^CH_4_ (99 atom % ^13^C; Sigma–Aldrich Co., St Louis, MO, USA), respectively, with six replicates. The ^12^CH_4_ and ^13^CH_4_ SIP treatments were incubated with a 60% maximum water-holding capacity and were maintained at 28°C in the dark throughout the incubation. When the headspace CH_4_ concentrations were reduced to < 1.40 ppmv in the SIP microcosms, destructive sampling was performed in triplicate for ^12^CH_4_ and ^13^CH_4_ treatments. The incubated soils were dug using a stainless-steel sampling spoon from each bottle and divided into subsamples. For nucleic acid extraction, 3.0 g of the soils was immediately suspended in RNA*later* (Ambion, Austin, TX, USA), stored at 4°C overnight, and frozen at −80°C. The remaining subsamples were stored at −20°C for further analysis.

The headspace gas in the remaining 10,000 ppmv CH_4_-amended microcosms was replaced with ambient air (∼1.86 ppmv CH_4_) to monitor the high-affinity CH_4_ oxidation activity. The microcosms were incubated at a 60% maximum water-holding capacity and 28°C in the dark throughout the incubation. The gas samples were immediately analyzed when the bottles were closed with gas-tight butyl rubber stoppers (hour 0). After 3 h of incubation, the gas samples were immediately analyzed (hour 3). The high-affinity CH_4_ oxidation activity was determined by calculating the amount of atmospheric CH_4_ that can be oxidized in the first 3 h (Cai et al., 2016).

CH_4_ concentration in the headspace was measured using a gas chromatography-flame ionization detector (GC-FID) (Shimadzu GC12-A, Kyoto, Japan). The column oven, injection, and FID detector temperatures were 40, 75, and 250, respectively. The flow rate of the carrier gas (N_2_) was 30 mL min^–1^. The injection volume was 200 μL, and the samples were analyzed twice. The equipment was calibrated as previously described (Preuss et al., 2013). CH_4_ standard gases at concentrations of 1.7, and 200 ppmv, 1, 10, and 50 vol % were used. The uncertainty due to manual injection onto the column was < 10% for the 1.7 ppmv standard and < 1% for the standards above 200 ppmv.

### 2.4 Nucleic acid extraction and SIP gradient fractionation

Total nucleic acids were extracted from paddy soils using the protocol developed by Mettel et al. (2010), with slight modifications (Cai et al., 2016). Soil samples stored at −80 °C in RNA*later* were thawed on ice. Subsequently, the samples were pelleted at 20,000 × *g* for 1 min to remove the supernatants. The pellets were mixed with 0.5 g of glass beads (0.5 mm: 0.1 mm = 3:2) and resuspended in acidic lysis buffers. The mixture was shaken using two rounds of bead-beating. The supernatant was obtained at 20,000 × *g* for 1 min, and successively extracted using water-saturated phenol (pH 4.5), phenol-chloroform–isoamyl alcohol (25:24:1 [vol/vol/vol], pH 4.5), and chloroform–isoamyl alcohol (24:1 [v/v], pH 5.5). The resulting aqueous phase was mixed with two volumes of PEG–NaCl (30% PEG-6000, 1.6 M NaCl). After incubation at 25°C for 5 min, the mixture was centrifuged at 20,000 × *g* for 30 min to obtain the nucleic acid pellet. The pellets were washed with 400 μL of 70% ethanol and resuspended in 50 μL of nuclease-free H_2_O. DNA was isolated from the total nucleic acid through RNase I digestion (Ambion, Austin, TX, USA). RNA was separated from the total nucleic acid through DNase I digestion (Ambion, Austin, TX, USA) and purified using an RNeasy Mini Kit (Qiagen, Hilden, Germany). Contaminating DNA in the RNA samples was assessed through PCR for 16S rRNA genes (Cai et al., 2016).

The quantity and purity of the nucleic acid were assessed using NanoDrop ND-1000 spectrophotometer (NanoDrop Technologies, USA). The purity of nucleic acid was represented by the absorbance ratio between nucleic acid (260 nm) and both humic acids and salts (230 nm) (A260/A230) and between nucleic acid (260 nm) and both humic acids and proteins (280 nm) (A260/A280). The DNA and RNA levels were in the range of 2.61–22.4 and 0.58–8.43 μg g^–1^ dry weight soil (*d.w.s*.) for the nine soils, respectively (Supplementary Table 2). The A260/A230 and A260/280 for DNA were in the range of 1.19–1.59 and 1.50–1.94, respectively. The A260/A230 and A260/280 for RNA were in the range of 1.14–1.62 and 1.48–1.87, respectively.

DNA-SIP (Zheng et al., 2014) and RNA-SIP fractionation (Dumont et al., 2011) in the ^12^CH_4_ and ^13^CH_4_ treatments were performed. RNA was reverse-transcribed into complementary DNA (cDNA) using a PrimeScript 1st Strand cDNA Synthesis Kit (Takara, Beijing, China) and random hexamers (Cai et al., 2016).

### 2.5 Real-time quantitative PCR of the *pmoA* genes

Total DNA from day 0 and ^13^CH_4_ treatment and fractionated DNA from the ^12^CH_4_ and ^13^CH_4_ treatments were selected for the real-time quantitative PCR (qPCR) of the *pmoA* genes using the primer pair A189F/mb661r (Costello and Lidstrom, 1999) on a CFX96 Optical Real-Time Detection System (Bio-Rad, Hercules, CA, USA). Real-time qPCR was performed as described previously (Zheng et al., 2014). The cycling conditions were set as follows: 3 min at 95°C, followed by 40 cycles of 95°C for 10 s, 55°C for 30 s, 72°C for 30 s, and 80°C for 5 s with the plate read. The melt curve analysis was monitored from 65 to 95°C. One representative cloning containing *pmoA* genes was used to generate standards. The plasmid DNA was extracted from the clone and then diluted to create a series of standard templates. The standard curve of bacteria *pmoA* genes covered 10^2^ to 10^8^ copies of template per assay. The amplification efficiencies for the *pmoA* genes were 93–99%, with R^2^ values of 0.991–0.999. A serial dilution of the DNA template in paddy soils was performed to assess whether the PCR was inhibited during the amplification. For quantifying the *pmoA* gene in the paddy soils, the fractionated DNA was undiluted, and the total DNA was diluted by 20-fold. The amplification specificity was investigated using melting curve analysis and standard agarose gel electrophoresis at the end of a PCR run.

### 2.6 High-throughput sequencing of the *pmoA* genes

Total DNA from day 0 and ^13^CH_4_ treatment and fractionated ^13^C-DNA containing the peak *pmoA* gene copies from the heavy fraction of the ^13^CH_4_ treatment were selected for high-throughput sequencing of *pmoA* genes using the barcode primer pair A189F/mb661r (Costello and Lidstrom, 1999) on a Roche 454 GS FLX Titanium sequencer (Roche Diagnostics Corporation, Branford, CT, USA). Raw sequence files were processed using the mothur software for quality control (Schloss et al., 2009). Low-quality sequence reads (read with lengths < 200 bp, ambiguous bases > 0, homopolymers > 6, primer mismatches, and average quality scores < 30) were removed. Subsequently, these sequences were processed with the online version of FunGene pipelines to remove the chimera using USEARCH 6.0 (Cai et al., 2016). The *pmoA* gene sequences were classified using a naïve classifier implemented with the mothur software (Zhao et al., 2020).

### 2.7 High-throughput sequencing of 16S rDNA and rRNA

Total DNA and cDNA from day 0 and ^13^CH_4_ treatment and fractionated DNA and cDNA from ^12^CH_4_ and ^13^CH_4_ treatments were selected for sequencing the 16S rDNA and rRNA, respectively, using the barcode primer pair 515F/907R (Zheng et al., 2014) on a Roche 454 GS FLX Titanium sequencer (Roche Diagnostics Corporation, Branford, CT, USA). Raw sequence files were processed using the mothur software for quality control (Schloss et al., 2009). Low-quality sequence reads (read with lengths < 200 bp, ambiguous bases > 0, homopolymers > 6, primer mismatches, and average quality scores < 30) were filtered for quality. Subsequently, the UCHIME algorithm was used to remove the chimeric sequences with a chimera-free reference database using the USEARCH tool (Ren et al., 2015). High-quality 16S rDNA and rRNA sequences were taxonomically classified using the Ribosomal Database Project classifier (Cai et al., 2016).

### 2.8 Statistical analysis

The methanotrophic communities in the paddy soils were compared using a one-way analysis of variance, followed by Tukey’s *post-hoc* test (*p* < 0.05). Statistical analyses were performed using SPSS version 24.0 (IBM Corporation, Armonk, NY, USA). Soil microbial co-occurrence network structures were constructed based on 16S rRNA sequencing data in R software using the SpiecEasi package (Cao et al., 2022) and visualized using the Gephi interactive platform (Barberán et al., 2012). Spearman’s rank correlations were calculated at the genus level for all taxa in the paddy soils on day 0 and when the soils can oxidize atmospheric CH_4_. Correlations with ρ > 0.6 and *p* < 0.05 were considered robust correlations (Gao et al., 2022). A redundancy analysis (RDA) was applied to investigate the influence of abiotic factors on the methanotrophic community composition in the paddy soils. Methanotrophic community composition was based on the *pmoA* gene in the paddy soils with high-affinity methane oxidation activity. The abundance of methanotrophic genera was expressed as the *pmoA* gene copies of total methanotrophs based on qPCR multiplied by the relative abundance of the targeted methanotrophic genera-related *pmoA* genes based on high-throughput sequencing. Detrended correspondence analysis (DCA) was used to analyze the data matrix, suggesting that the best-fit mathematical model was the RDA. Permutational multivariate analysis of variance (PERMANOVA) was used to test whether the different abiotic factors harbored significantly different methanotrophic communities (Kaupper et al., 2022). The RDA analysis was implemented in the R package vegan v4.2.1 function.

## 3 Results

### 3.1 High-affinity oxidation of atmospheric CH_4_ in paddy soils

CH_4_ could not be oxidized under conditions involving 2 or 100 ppmv concentrations in the microcosms (Figure 1B). In contrast, the headspace CH_4_ concentrations were significantly reduced during the incubation with CH_4_ concentrations of 1,000 and 10,000 ppmv. CH_4_ concentrations in the 10,000 ppmv CH_4_-amended microcosms reduced to < 1.40 ppmv for 4–11 days in paddy soils (Figure 1B), indicating the widespread potential to use atmospheric CH_4_ in paddy soils.

**FIGURE 2:**
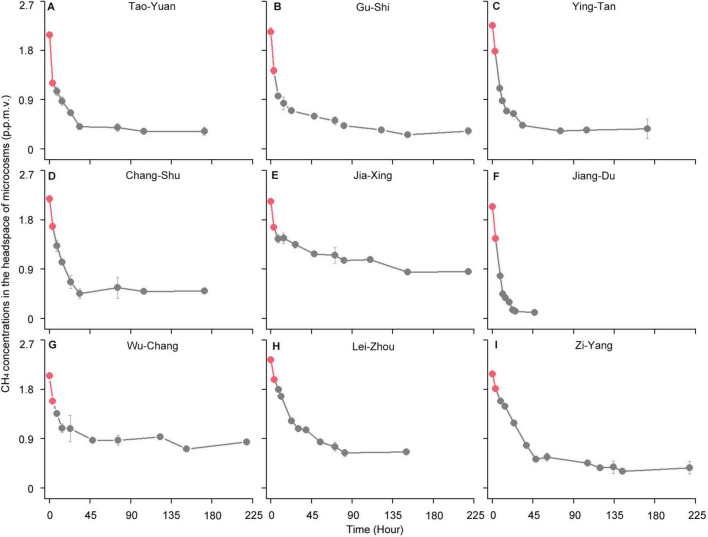
High-affinity methane (CH_4_) oxidation dynamics of paddy soils under atmospheric CH_4_ concentrations **(A–I)**. **(A)** Tao-Yuan; **(B)** Gu-Shi; **(C)** Ying-Tan; **(D)** Chang-Shu; **(E)** Jia-Xing; **(F)** Jiang-Du; **(G)** Wu-Chang; **(H)** Lei-Zhou; **(I)** Zi-Yang. After the complete depletion of 10,000 parts per million by volume (ppmv) CH_4_, the headspace of microcosms is renewed with ambient air (∼1.86 ppmv CH_4_). The circles indicate time points for analyzing CH4 concentrations in the microcosms. The two red circles for each soil sample represent time points at 0 and 3 h, respectively. The amount of atmospheric CH4 oxidized in the first 3 h was used to assess the high-affinity CH_4_ oxidation activity, as described in Supplementary Table 3. The error bars represent standard deviations of triplicate microcosms.

Following the consumption of 10,000 ppmv CH_4_, the headspace gas of the microcosms was renewed with ambient air (∼1.86 ppmv CH_4_) (Figure 2). The atmospheric CH_4_ concentrations were rapidly reduced in all paddy soils. The high-affinity CH_4_ oxidation activity, assessed based on the amount of atmospheric CH_4_ oxidized in the first 3 h, varied from 0.07 to 0.23 nmol of CH_4_ h^–1^ g^–1^ dry weight soil (Supplementary Table 3). However, the headspace CH_4_ concentrations in the 1,000 ppmv CH_4_-amended microcosms did not reduce to < 1.86 ppmv in all paddy soils except in Lei-Zhou (Figure 1B). These results indicate that the threshold of CH_4_ concentrations that induce high-affinity CH_4_ oxidation activity for atmospheric CH_4_ varies among different paddy soils.

### 3.2 Active methanotrophs responsible for high-affinity CH_4_ oxidation in paddy soils

Following the consumption of 10,000 ppmv CH_4_, the paddy soils possessed high-affinity CH_4_ oxidation activity (Figure 1B). The paddy soils were collected and then used to investigate methanotrophic community based on a high-throughput sequencing of *pmoA* genes, 16S rDNA, and 16S rRNA (Supplementary Tables 4, 5). Compared with the soils on day 0, a substantial growth of total methanotrophs was observed in all paddy soils with high-affinity CH_4_ oxidation activity (Supplementary Figures 1A–C).

**FIGURE 3:**
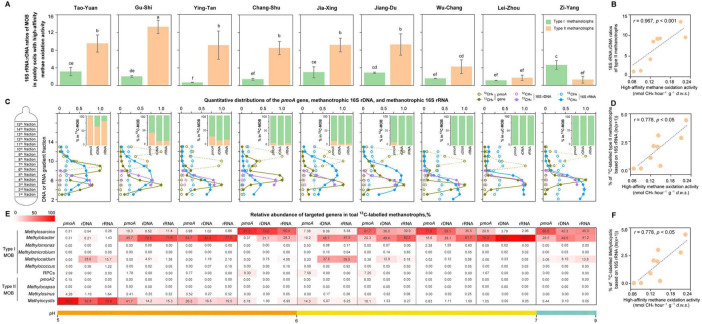
Activity of methanotrophs by assessing the 16S rRNA:16S rDNA ratios and documenting the relative abundance of ^13^C-labeled methanotrophs in paddy soils. **(A)** The ratios of 16S rRNA to 16S rDNA of methanotrophic subgroups (type I and type II) in paddy soils with high-affinity CH_4_ oxidation activity. The 16S rRNA:rDNA ratios of methanotrophs are expressed as the ratios of the relative abundance of targeted methanotrophic 16S rRNA in total 16S rRNA to the relative abundance of targeted methanotrophic 16S rDNA in total 16S rDNA based on high-throughput sequencing in each sample. The error bars represent standard deviations of triplicate microcosms. Different letters above the columns indicate a significant difference (analysis of variance [ANOVA], *p* < 0.05). **(B)** Relationship between high-affinity CH_4_ oxidation activity and the 16S rRNA:16S rDNA ratios of type II methanotrophs in paddy soils (*n* = 9). There is no significant correlation between high-affinity CH_4_ oxidation activity and the 16S rRNA:rDNA ratios of type I methanotrophs. Statistical analysis is performed using Spearman’s rank-based correlation. **(C)** Quantitative distributions of the *pmoA* gene, methanotrophic 16S rDNA, and methanotrophic 16S rRNA in paddy soils with high-affinity CH_4_ oxidation activity. ^13^CH_4_ and ^12^CH_4_ represent the paddy soils incubated with 10,000 parts per million by volume (ppmv) ^12^CH_4_ and 10,000 ppmv ^13^CH_4_, respectively. The *pmoA* gene and methanotrophic 16S rDNA are across the buoyant density gradient of the DNA fractions, and methanotrophic 16S rRNA is across the buoyant density gradient of the RNA fractions. For *pmoA* genes, the normalized data are the ratios of gene copy number in each DNA fraction to the maximum quantities from each treatment based on real-time quantitative PCR. The abundance of methanotrophs based on high-throughput sequencing is expressed as the proportion of methanotrophic 16S rDNA to the total 16S rDNA in each DNA fraction and the proportion of methanotrophic 16S rRNA to the total 16S rRNA in each RNA fraction. The pentagrams in olive, purple, and blue indicate the ^13^C-labeled *pmoA* genes, ^13^C-labeled 16S rDNA, and ^13^C-labeled 16S rRNA that are used for the analysis of active methanotrophic community composition using high-throughput sequencing, respectively. The columns indicate the ^13^C-labeled methanotrophic community composition that is expressed as the percentage of the targeted methanotrophic subgroup to the total ^13^C-labeled methanotrophs based on the ^13^C-*pmoA* gene, ^13^C-16S rDNA, and ^13^C-16S rRNA. **(D)** Relationship between high-affinity CH_4_ oxidation activity and relative abundance of ^13^C-labeled type II methanotrophs based on 16S rRNA in paddy soils (*n* = 9). The correlations between high-affinity CH_4_ oxidation activity and the relative abundance of ^13^C-labeled methanotrophs based on the *pmoA* gene and 16S rDNA are also significant and positive, as described in Supplementary Table 6. The relative abundance of ^13^C-labeled methanotrophs is log-transformed before statistical testing. Statistical analysis is performed using Spearman’s rank-based correlation. **(E)** The community composition of ^13^C-labeled methanotrophs at the genus level. The numbers indicate the percentage of the targeted methanotrophic genus to total methanotrophs based on the ^13^C-labeled *pmoA*, ^13^C-labeled 16S rDNA, and ^13^C-labeled 16S rRNA. **(F)** Relationship between high-affinity CH_4_ oxidation activity and the relative abundance of ^13^C-labeled *Methylocystis* based on 16S rRNA in paddy soils (*n* = 9). The correlations between high-affinity CH_4_ oxidation activity and the relative abundance of ^13^C-labeled *Methylocystis* based on the *pmoA* gene and 16S rDNA are also significant and positive (Supplementary Table 6). The relative abundance of ^13^C-labeled *Methylocystis* is log-transformed before statistical testing. Statistical analysis is performed using Spearman’s rank-based correlation.

The 16S rRNA:rDNA ratios of type I methanotrophs varied in different paddy soils with high-affinity CH_4_ oxidation activity, as well as type II methanotrophs (Figure 3A). The 16S rRNA:rDNA ratios of type II methanotrophs were 1.27–12.5-fold higher than those of type I methanotrophs in each paddy soil with high-affinity CH_4_ oxidation activity, except in Zi-Yang. In contrast, 16S rRNA:rDNA ratio of type II methanotrophs in the alkaline paddy soil in Zi-Yang, was 3.61-fold lower than that of type I methanotrophs. Notably, the eight paddy soils, possessing higher 16S rRNA:rDNA ratios for type II methanotrophs than that for type I methanotrophs, exhibited acid-neutral soils with a pH of 5.18–6.80. This indicates the higher potential activity of type II methanotrophs compared to that of type I methanotrophs in high-affinity CH_4_ oxidation in acid-neutral paddy soils. We observed a positive correlation between the 16S rRNA:rDNA ratios of type II methanotrophs and high-affinity CH_4_ oxidation activity (Figure 3B) but not for type I methanotrophs (Supplementary Table 6). The 16S rRNA:rDNA ratios of type II methanotroph *Methylocystis* were higher than those of any type I methanotrophic genus in each acid-neutral soil (Supplementary Figure 2A). Additionally, we observed a significant positive relationship between the 16S rRNA:rDNA ratios of type II methanotroph *Methylocystis* and high-affinity CH_4_ oxidation activity in paddy soils (Supplementary Figure 2B).

Additionally, 10,000 ppmv ^13^CH_4_ can target active methanotrophs, which can help in exploring the relative role of individual methanotrophic taxa in soil CH_4_ oxidation (Figure 3C). The highly enriched *pmoA* gene, methanotrophic 16S rDNA, and methanotrophic 16S rRNA were observed in heavy fractions from the ^13^CH_4_ treatment (Figure 3C and Supplementary Tables 7, 8). It indicates that both the genomes and transcriptomes of methanotrophic cells were strongly labeled in the ^13^CH_4_-treated soils. ^13^C-labeled 16S rRNA reads revealed a significantly higher proportion of type I methanotrophs than that of type II methanotrophs in each paddy soil, except in the most acidic soil, in Tao-Yuan (Figure 3C). A similar trend, a higher proportion of type I methanotrophs compared to that of type II methanotrophs, was observed using ^13^C-labeled *pmoA* genes and 16S rDNA in these eight paddy soils. The results indicate that type II methanotrophs displayed a lower growth rate compared with type I methanotrophs during the low-affinity oxidation of 10,000 ppmv high-CH_4_ in most paddy soils. However, we observed a significant positive relationship between the relative abundance of ^13^C-labeled type II methanotrophs and high-affinity CH_4_ oxidation activity (Figure 3D) but not for ^13^C-labeled type I methanotrophs (Supplementary Table 6).

Almost all type II methanotrophs were phylogenetically related to *Methylocystis* in the paddy soils (Figure 3E). Although ^13^C-labeled *Methylocystis* constituted a lower fraction of methanotrophs than the type I methanotrophic genera (*Methylosarcina*, *Methylobacter*, or *Methylocaldum*), it was positively correlated with high-affinity CH_4_ oxidation activity in paddy soils (Figure 3F). We also observed a positive correlation between the high-affinity CH_4_ oxidation activity and *pmoA* gene copy numbers of *Methylocystis* (Supplementary Table 6). For type II methanotroph *Methylosinus*, a very low abundance was detected in all paddy soils (Figure 3E); however, it was positively correlated with the high-affinity CH_4_ oxidation activity (Supplementary Table 6).

### 3.3 Biotic interactions of methanotrophs and other prokaryotic taxa

**FIGURE 4:**
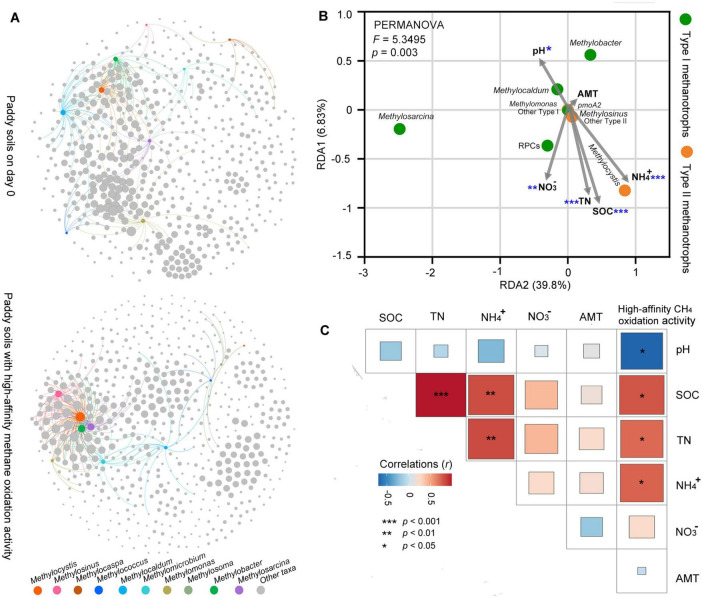
Biotic interactions of methanotrophs and other prokaryotic taxa, and abiotic factors and their association with methanotrophic community and high-affinity methane oxidation activity in paddy soils. **(A)** Co-occurrence network analysis demonstrating the associations among all prokaryotic genera in the paddy soils. The nodes represent the prokaryotic microorganisms at the genus level based on 16S rRNA. Links between the nodes indicate a significant correlation (correlation > 0.6, *p* < 0.05). For visual clarity, only links among methanotrophs-other prokaryotic taxa are illustrated. The bigger the node, the higher the degree (the number of network links for the node). Details of the network among all prokaryotic genera are listed in Supplementary Figure 4. **(B)** Redundancy analysis (RDA) showing the abiotic factors (pH, SOC, TN, NH_4_^+^, NO_3_^–^, and AMT) affecting the methanotrophic community as constraints. Significant abiotic factors affecting the methanotrophic community composition are marked with asterisks (*p < 0.05; **p < 0.01; ***p < 0.001). **(C)** Correlations between abiotic factors and high-affinity methane oxidation activity. Statistical analysis is performed using Pearson’s rank-based correlation (*n* = 9). The abbreviation AMT represents annual mean temperature in the soil sampling site. All other abbreviations are the same as those in Figure 1.

Co-occurring network analysis indicated that the number of links (degree) among methanotrophs and other prokaryotic taxa was higher in the paddy soil with high-affinity CH_4_ oxidation activity (degree = 227) than in the paddy soils on day 0 (degree = 162) (Figure 4A and Supplementary Figure 4A). This indicates that atmospheric CH_4_ oxidation enhanced the possible biotic interactions between methanotrophs and other prokaryotic taxa in the paddy soils. The significant correlation between methanotroph and prokaryotic taxa involved in the nitrogen cycle, such as diazotrophs (e.g., *Bradyrhizobium*, *Burkholderia*, *Acidisoma*, and *Mesorhizobium*), and nitrifying bacteria (e.g., *Nitrobacter*, and *Nitrospira*), was observed during CH_4_ oxidation (Supplementary Figure 4B). Notably, *Methylocystis* exhibited the highest degree in the network when the paddy soil could oxidize atmospheric CH_4_ (Figure 4A).

### 3.4 Abiotic factors and their association with methanotrophic community and high-affinity methane oxidation activity in the paddy soils

The RDA integrated the abiotic factors to methanotrophic community composition in the paddy soils with high-affinity methane oxidation activity (Figure 4B). Among the abiotic factors, soil pH and soil nutrient availability (SOC, TN, NH_4_^+^, and NO_3_^–^) significantly affected the methanotrophic community composition (Figure 4B). The abundant type I methanotrophs (*Methylosarcina*, *Methylobacter*, *Methylocaldum*, and rice paddy clusters [RPCs]) and type II methanotrophs (*Methylocystis*, and *Methylosinus*) exhibited different responses to environmental factors. Type II methanotrophs were commonly found in more acidic paddy soils compared to type I methanotrophs. The abundance of type II methanotrophs, especially *Methylocystis*, were positively correlated to soil nutrient availability (SOC, TN, and NH_4_^+^) in the paddy soils, whereas type I methanotroph RPCs were positively correlated with soil NO_3_^–^. The high-affinity methane oxidation activity exhibited a negative correlation with soil pH and a positive correlation with nutrient availability (SOC, TN, and NH_4_^+^) (Figure 4C), similar to the correlations between abiotic factors and type II methanotrophs. Contrary to our expectations, the annual mean temperature had no significant effect on the methanotrophic community composition (Figure 4B) or high-affinity methane oxidation (Figure 4C).

## 4 Discussion

Wetlands are typically considered CH_4_ sources (IPCC, 2021). However, some periodically drained wetland ecosystems, such as the flooded paddy soils (Conrad, 2007), Arctic wetlands (Voigt et al., 2023), and mire-wetlands (Wang et al., 2023), serve as CH_4_ sinks during the dry periods. In this study, we selected paddy soils across three climate zones from the primary rice production areas in China to obtain extensive spatial coverage of atmospheric CH_4_ uptake in paddy soils.

The CH_4_ concentrations in flooded rice fields vary with alterations in water content (Conrad, 2007). We constructed identical microcosms with various initial CH_4_ concentrations to assess the occurrence of high-affinity CH_4_ oxidation in periodically draining paddy soils, where CH_4_ availability fluctuated. All tested paddy soils can oxidize atmospheric CH_4_ following the consumption of CH_4_ at high concentrations (10,000 ppmv) (Figure 1B). The estimated atmospheric CH_4_ consumption rate in paddy soils varied from 1.87 to 6.03 kg CH_4_ ha^–1^ per year (Supplementary Table 3), consistent with the values reported for soil ecosystems (Aronson et al., 2013). Our experiments indicate that paddy soils can serve as a CH_4_ sink if drained after periodic flooding in rice fields with high concentrations of CH_4_.

In contrast to microcosms with 10,000 ppmv of CH_4_, we did not observe high-affinity oxidation of atmospheric CH_4_ in all paddy soils incubated with CH_4_ at concentrations ranging from 2 to 1,000 ppmv, except in Lei-Zhou soil incubated with 1,000 ppmv CH_4_ (Figure 1B). A threshold amount of CH_4_ consumption for the induction of atmospheric CH_4_ uptake may vary greatly among different soils; however, the underlying mechanism remains elusive (Cai et al., 2016). The lower threshold in Lei-Zhou could not be attributed to the abiotic and biotic factors noted in our study. We hypothesize that it may be explained by the influence of unmeasured environmental factors. For instance, soil texture can influence gas diffusion and soil aeration (Wang et al., 2022). The gas diffusion may regulate the CH_4_ and O_2_ availability and methanotrophic activity (Lee et al., 2023). Hence, further consideration of more soil factors is needed to identify the underlying mechanisms.

The ribosome content per cell follows bacterial growth (Kemp et al., 1993). rDNA can be extracted from living, dormant, and dead microbial cells (Josephson et al., 1993). rRNA is generally positively correlated with the growth rate of bacteria and degrades during certain stress conditions, such as substrate starvation (Deutscher, 2006). The 16S rRNA:rDNA ratio serves as an indicator of bacterial taxa activity in natural communities (Campbell et al., 2011; Campbell and Kirchman, 2013; Lankiewicz et al., 2016). The 16S rRNA:rDNA ratio is more informative than abundance alone in understanding the microbial activity in the environment (Campbell and Kirchman, 2013). The 16S rRNA:rDNA ratio of methanotrophs indicated that type II methanotrophs exhibited higher potential activity than type I methanotrophs during high-affinity CH_4_ oxidation in most paddy soils (Figure 3A). Additionally, 16S rRNA:rDNA ratios of type II methanotrophs positively correlated with the high-affinity CH_4_ oxidation activity (Figure 3B).

Notably, significant positive relationships were observed between the 16S rRNA:rDNA ratios and potential growth rates, as determined by assessing the relative abundance of ^13^C-labeled methanotrophs for type II methanotrophs (Supplementary Figure 3). This indicates that type II methanotrophs can survive under CH_4_-starvation conditions without markedly rRNA degradation. Although type II methanotrophs was not found to dominate in most paddy soils (Figure 3C), its abundance was positively correlated with high-affinity methane oxidation activity (Figure 3D). This finding was particularly noted *Methylocystis* (Figure 3F). Some rare taxa may exhibit higher microbial activity compared with the abundant taxa in the environment (Campbell et al., 2011). These findings support a better adaptation of type II methanotrophs, especially *Methylocystis*, to atmospheric CH_4_ than that of type I methanotrophs in paddy soils.

High-affinity CH_4_ oxidation activity is due to the consumption of high concentrations of CH_4_ in paddy soils (Yan and Cai, 1997; Cai et al., 2016). Theoretically, atmospheric CH_4_ does not provide adequate energy for the survival of cultured methanotrophs (Dunfield, 2007). High-affinity methanotrophs living in atmospheric CH_4_ can obtain energy from additional sources (Baani and Liesack, 2008; Cai et al., 2016; Tveit et al., 2019). Endogenous storage compounds that have accumulated during exposure to high CH_4_ concentrations, such as polyhydroxybutyrate (PHB), can potentially provide reductive power for CH_4_ monooxygenase in methanotrophs during atmospheric CH_4_ oxidation (Pieja et al., 2011; Cai et al., 2016). PHB production has been observed in type II methanotrophs, such as *Methylocystis* and *Methylosinus*, whereas type I methanotrophs may not be able to produce PHB (Pieja et al., 2011). Culturable methanotrophs sustain atmospheric methane oxidation if supplied with formate (Jensen et al., 1998). Formate oxidation reaction could provide the donor electron for particulate methane monooxygenase (pMMO) to sustain high-affinity methane oxidation (Le and Lee, 2023). Type II methanotrophs can transform acetate to acetyl-CoA (Singleton et al., 2018). Acetate as a source of carbon and energy allows methanotrophs to maintain methane oxidation activity under CH_4_ starvation conditions (Belova et al., 2011; Singleton et al., 2018). Some type II methanotrophs harvest additional energy from aerobic respiration of hydrogen (H_2_) at atmospheric concentrations (Tveit et al., 2019).

In addition to the known type of pMMO1 responsible for high CH_4_ concentrations, most type II methanotrophs possess pMMO2 to oxidize CH_4_ at atmospheric concentrations (Baani and Liesack, 2008). However, pMMO2 may not be detected in any known type I methanotrophs (Tchawa Yimga et al., 2003). In this study, *pmoA2* genes that encode pMMO2 were observed in the acid-neutral paddy soils (Jiang-Du, Gu-Shi, Ying-Tan, Tao-Yuan, and Chang-Shu) that can oxidize atmospheric CH_4_ (Supplementary Table 4). Moreover, the type II methanotroph *Methylocapsa*, whose ^13^C-labeled 16S rRNA was observed in Tao-Yuan (Figure 3E), encoded a single PMMO responsible for CH_4_ oxidation at high and atmospheric concentrations (Tveit et al., 2019). The coexistence of high- and low-affinity CH_4_ oxidation activities may be advantageous for type II methanotrophs to thrive in paddy soils, where CH_4_ concentrations fluctuate significantly. Therefore, in type II methanotrophs, the capacity for PHB production, acetate and hydrogen uptake, and high-affinity pMMO expression provides a selective advantage for survival under CH_4_ starvation conditions in paddy soils.

Type I methanotrophs may also play a role in the high-affinity CH_4_ oxidation, specifically in the alkaline Zi-Yang soil (pH 8.23). The 16S rRNA:rDNA ratio (Figure 3A) and the abundance (Figure 3C) were remarkably higher for type I methanotrophs than that for type II methanotrophs in Zi-Yang. Numerous strains within type I methanotrophs can oxidize atmospheric CH_4_ after incubation with high CH_4_ concentrations (Schnell and King, 1995; Benstead et al., 1998). Type I methanotrophic *pmoA* transcripts are observed in a paddy soil with atmospheric CH_4_ oxidation activity (Cai et al., 2016).

CH_4_ oxidation enhanced a complex network of microbial interactions among the methanotrophs and other prokaryotic taxa, as illustrated in Figure 4A. In addition, linkages between *Methylocystis* and other taxa occurred at the highest proportion in the paddy soils with high-affinity CH_4_ oxidation activity (Figure 4A). Organic carbon derived from a source of CH_4_ via the assimilation of methanotrophs facilitates the growth of non-methanotrophs (Kaupper et al., 2022). For instance, type II methanotrophs *Methylocystis* and *Methylosinus* were positively correlated with *Methylobacterium* (Supplementary Figure 4B). *Methylobacterium* can use methanol as an energy and carbon source (Tani et al., 2021). Cross-feeding might drive their positive correlations via the methanol from the oxidation of methane by methanotrophs. In addition, methanotrophs-nitrifying bacteria and methanotrophs-diazotrophs interactions linked carbon and nitrogen cycling in paddy soils (Supplementary Figure 4B). Nitrifying bacteria are chemoautotrophs (Xia et al., 2011), and CH_4_-derived CO_2_ can be incorporated by chemoautotrophs (Kaupper et al., 2022). Diazotrophs can contribute reactive nitrogen to natural ecosystems (Hu et al., 2024). The available nitrogen from diazotrophs might drive the positive links between diazotrophs and *Methylocystis*.

The methanotrophic community (Figure 4B) and methane oxidation uptake (Figure 4C) respond to various abiotic factors in the paddy fields, such as CH_4_ content, pH, nutrient availability and temperature. The variations in soil moisture attributed to periodic drainage in rice fields could result in significant fluctuations in soil CH_4_ concentrations (Conrad, 2007). The rice fields are similar to aerated upland soils during the dry seasons (Conrad, 2007). Our paddy soils were collected in the drained rice fields, where CH_4_ availability was limited. The dominance of type II methanotrophs on day 0 (Supplementary Figure 1D) aligns with a K-type life strategy, characterized by an investment in survival and longevity (Steenbergh et al., 2010; Ho et al., 2013). This strategy may be advantageous for type II methanotrophs that can occupy niches under resource limiting conditions. It helps explain why the type II methanotrophic activity (based on 16S rRNA:rDNA ratios) was higher than that of type I methanotrophs during atmospheric methane oxidation in most paddy soils (Figure 3A). We incubated the paddy soils with CH_4_ at a concentration of 10,000 ppmv to mimic the high availability of CH_4_ noted under flooded conditions. The faster growth of type I methanotrophs in most paddy soils (Figure 3C) is an indication of an r-type life strategy that emphasizes high reproductive success, which is instantaneous under favorable conditions (Qiu et al., 2008; Steenbergh et al., 2010). Type I and II methanotrophs possess distinct life strategies, offering them a selective advantage under various environmental conditions (Ho et al., 2013).

Soil pH significantly regulated methanotrophic community composition (Figure 4B) and negatively influenced CH_4_ uptake (Figure 4C) in paddy soils. To date, most known type II methanotrophs cannot survive above pH 8.0; however, cultivated type I methanotrophs are more tolerant to high pH conditions than type II methanotrophs (Kalyuzhnaya et al., 2019; Yao et al., 2022). This finding helps explain why higher 16S rRNA:rDNA ratios for type I methanotrophs, compared to type II methanotrophs, were only observed in the alkaline paddy soil of Zi-Yang (pH 8.23) under methane-starvation conditions (Figure 3A). A majority of acidophilic methanotrophs are type II (Hwangbo et al., 2023). In paddy soils with pH values ranging from 5.0 to 8.0, type II methanotrophs are more widespread in low pH conditions than in high pH conditions (Shiau et al., 2018; Zhao et al., 2020). A pH value of less than 5.2 is a key driving force for the selection of type II over type I methanotrophs in paddy soils at a high-methane condition (Shiau et al., 2018). It helps explain that faster growth of type II than type I methanotrophs during the low-affinity oxidation of high-methane was only detected in the acidic soil Tao-Yuan (pH 5.18) (Figure 3C). Type II methanotrophs had positive effect on atmospheric CH_4_ take (Figures 3B, D). Soil pH was negatively correlated with the abundance of type II methanotrophs, especially *Methylocystis* (Figure 4B), thereby, influencing the atmospheric CH_4_ uptake (Figure 4C). A high influence of soil pH on atmospheric CH_4_ uptake was also observed in Arctic soils, where CH_4_ uptake increased with lower soil pH (Voigt et al., 2023).

Soil nutrient availability, such as SOC, TN, and NH_4_^+^, positively affected the abundance of type II methanotrophs, especially *Methylocystis* (Figure 4B), and atmospheric CH_4_ uptake (Figure 4C) in paddy soils. Higher SOC content results in increased atmospheric CH_4_ uptake in forest soils (Lee et al., 2023). SOC increases the soil pore space and, therefore, diffuses atmospheric CH_4_ into the soil (Lee et al., 2023). In addition, SOC can facilitate atmospheric methanotrophic activity by providing alternative carbon substrates, such as formate and acetate, during CH_4_ starvation (Jensen et al., 1998, West and Schmidt, 1999, Lee et al., 2023). The capability of using acetate as a carbon and energy source is a common trait in *Methylocystis* (Belova et al., 2011; Singleton et al., 2018). It may be advantageous for *Methylocystis* under CH_4_-limited conditions.

Nitrogen interacts with atmospheric methane uptake in the soil ecosystems (Bodelier and Steenbergh, 2014; Voigt et al., 2023). Nitrogen as a nutrient stimulates the growth of methanotrophs and promotes methanotrophic oxidation activity (Zheng et al., 2014; Peng et al., 2019; Nijman et al., 2021). Nitrogen is the main limiting nutrient for methanotrophs in rice fields (Bodelier and Steenbergh, 2014). Enhanced nitrogen promotes CH_4_ uptake in N-limiting soils (Bodelier and Steenbergh, 2014; Peng et al., 2019; Voigt et al., 2023). In contrast to type I methanotrophs, type II methanotrophs can fix atmospheric nitrogen to assimilable nitrogen forms (Ho et al., 2013). Therefore, type II methanotrophs possess an advantage when nitrogen is limited. Soil NO_3_^–^ content showed no significant effect on atmospheric CH_4_ uptake (Figure 4C). In addition to acting as a nutrient, NO_3_^–^ exerts a toxic effect that could inhibit methane uptake (Ho et al., 2013). It adds to the complexity of methane oxidation-nitrogen assimilation interactions.

CH_4_ sink is enhanced with increasing temperature in forest soils (Lee et al., 2023). However, the temperature has no significant effect on the composition of methanotrophic community (Figure 4B) and atmospheric oxidation activity (Figure 4C) in paddy soils. Temperature might influence atmospheric CH_4_ uptake via other soil variables (Lee et al., 2023; Lin et al., 2023). For instance, increased temperature accelerates soil organic carbon decomposition (Zhao et al., 2023; Nazir et al., 2024) and, therefore the methanotrophic activity (Lee et al., 2023). We cannot observe a significant correlation between the temperature and SOC content (Figure 4C), probably due to the influence of unmeasured environmental factors on SOC and methanotrophic activity.

Overall, we observed higher atmospheric CH_4_ oxidation activity in paddy soils with lower soil pH, higher nutrient availability (SOC, TN, and NH_4_^+^), and higher type II methanotrophic activity, especially *Methylocystis*. Abiotic factors could influence the methanotrophic community and, hence, the atmospheric CH_4_ oxidation activity. This large-scale study examining atmospheric CH_4_ sink in paddy soils will facilitate an understanding of the relationships between atmospheric CH_4_ uptake, environmental factors, and microbial community. These results help understand how biotic and abiotic factors affect the atmospheric CH_4_ uptake in paddy soils. Our study helps explore the potential farm management (e.g., field fertilization and irrigation strategies) to promote CH_4_ uptake in paddy soils, providing avenues to develop strategies to mitigate global climate change.

The large-scale field sites investigated here were located within a wide range of climates, soil conditions, and microbial communities; however, certain special conditions cannot be explained by the measured factors in our study. Therefore, it is important to consider the limitations of this study. (1) The threshold amount of methane consumption for the induction of high-affinity methane oxidation was lower in Lei-Zhou (1,000 ppmv) than in other soils (10,000 ppmv). However, the underlying mechanism remains elusive according to the measured biotic factors and abiotic factors. To understand the underlying mechanisms, we should investigate more detailed biotic factors via advanced methods, such as metatranscriptomics and metaproteomics, and analyze more abiotic factors (e.g., soil texture). (2) Increasing temperature can enhance atmospheric methane uptake in forest soils. However, temperature did not influence atmospheric methane uptake in paddy soils. The range of annual mean temperature was narrow in our study, in the range of 16–19°C in most sampling sites. In addition, temperature might influence the atmospheric CH_4_ uptake via unmeasured environmental factors. Therefore, more soil samples from a wider temperature range and more soil factors are needed to provide deeper mechanistic insights into the underlying mechanisms. (3) The higher activity of type I methanotrophs (the ratio of 16S rRNA:rDNA) in “high-affinity” soils was observed only in one alkaline soil. We should examine more soil samples to determine whether the trait is widespread in alkaline soils. (4) The selection of type II over type I methanotrophs was observed under high-methane conditions only in Tao-Yuan, which is not consistent with the life strategy for methanotrophs. Low pH condition (pH < 5.2) is the possible driving factor; however, the unknown potential environmental factors are of significant interest. More paddy soils, especially acidic soils, and more abiotic factors should be considered to refine our understanding of the underlying mechanisms in the future. Taken together, more paddy soils with broader environmental conditions (e.g., wider soil pH range and annual mean temperature range), more soil factors (e.g., soil texture), and more advanced methodologies for analyzing methanotrophic activity (e.g., metatranscriptomics and metaproteomics approaches) are needed to validate and expand upon our findings in the future.

## 5 Conclusion

High-affinity CH_4_ oxidation induced by high CH_4_ concentrations is widespread in paddy soils, as indicated by the large spatial coverage of atmospheric CH_4_ uptake measurements. In acid-neutral paddy soils capable of oxidizing atmospheric CH_4_, type II methanotrophs exhibited higher 16S rRNA:rDNA ratios and, therefore, higher potential activity than type I methanotrophs. Additionally, the methanotrophic activity was analyzed by examining the relative abundance of ^13^C-labeled methanotrophs using both DNA- and RNA-SIP. Significant positive relationships were observed between the high-affinity CH_4_ oxidation activity and 16S rRNA:rDNA ratios of type II methanotrophs and the relative abundance of ^13^C-labeled type II methanotrophs. The microbial co-occurrence network indicated that CH_4_ oxidation enhanced biotic interactions between methanotrophs and other prokaryotic taxa. Soil pH and nutrient availability can significantly affect the methanotrophic community and high-affinity CH_4_ oxidation activity. The abundance of type II methanotrophs, especially *Methylocystis*, as well as the high-affinity methane oxidation activity, showed a negative correlation with soil pH and a positive correlation with soil nutrient availability (SOC, TN and NH_4_^+^). Our results offer a wide biogeographical perspective on atmospheric CH_4_ uptake in paddy soils and help to explore the mitigation strategy for global climate change via optimal farm management (e.g., field fertilization and irrigation strategies) in paddy fields. Future research should investigate a broader range of paddy soil samples and soil factors to further elucidate the underlying mechanisms unidentified in our study, providing a deeper insight into greenhouse gas mitigation strategies.

## Data Availability

The raw sequence data reported in this article are available in the NCBI Sequence Read Archive under BioProject PRJNA1030075.
