# Supplementary material for: Role of methanotrophic communities in atmospheric methane oxidation in paddy soils
Source: Front Microbiol. 2024 Nov 6;15:1481044. doi: 10.3389/fmicb.2024.1481044 (PMC11578120; doi:10.3389/fmicb.2024.1481044)
Supplement: Supplementary file 1 [file Data_Sheet_1.docx]

**Supplementary information**

**The supplementary information includes:**

1. Supplementary Figure Legends 1 to 4
2. Supplementary Tables 1 to 8


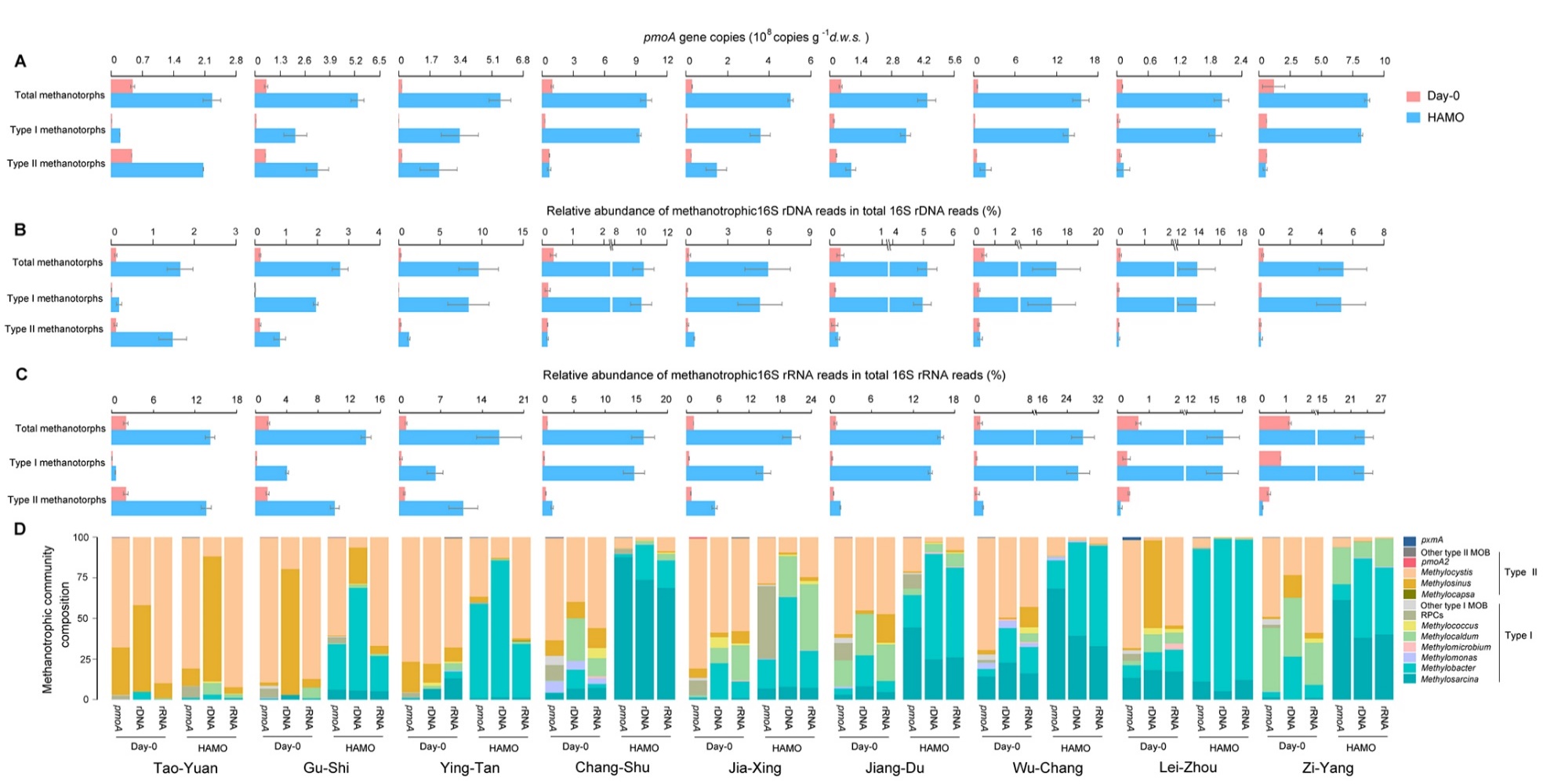


**Supplementary Figure 1 Changes in the methanotrophic community in paddy soils on day 0 and in paddy soils with high-affinity methane oxidation activity.** **(A**) Change in the *pmoA* gene copy number of paddy soils. The *pmoA* gene copies of total methanotrophs based on qPCR multiplied by the relative abundance of *pmoA* genes affiliated to the targeted subgroup based on high-throughput sequencing make the *pmoA* gene copy number of methanotrophic subgroups (type I and type II methanotrophs). ‘HAMO’ represents the 10,000 p.p.m.v. methane-amended paddy soils with high-affinity methane oxidation activity. The error bars represent the standard deviations of two replicates on Day 0 and triplicates in the paddy soil with high-affinity methane oxidation activity. **(B)** Changes in the relative abundance of methanotrophs in paddy soils based on high-throughput sequencing of 16S rDNA. The relative abundance of methanotrophs is expressed as the percentage of the targeted subgroup-related 16S rDNA to the total 16S rDNA in paddy soils. The error bars represent the standard deviations of two replicates on Day 0 and triplicates in the paddy soil with high-affinity methane oxidation activity. **(C)** Changes in the relative abundance of methanotrophs in paddy soils based on high-throughput sequencing of 16S rRNA. The relative abundance of methanotrophs is expressed as the percentage of the targeted subgroup-related 16S rRNA to the total 16S rRNA in paddy soils. The error bars represent the standard deviations of two replicates on Day 0 and triplicates in the paddy soils with high-affinity methane oxidation activity. **(D)** Change in the community composition of methanotrophs in paddy soils. ‘*pmoA*’, ‘rDNA’, and ‘rRNA’ represent the percentage of the targeted genus to total methanotrophs analyzed by high-throughput sequencing of the *pmoA* gene, 16S rDNA, and 16S rRNA, respectively. For clarity, the standard error bar from the replicates was removed.


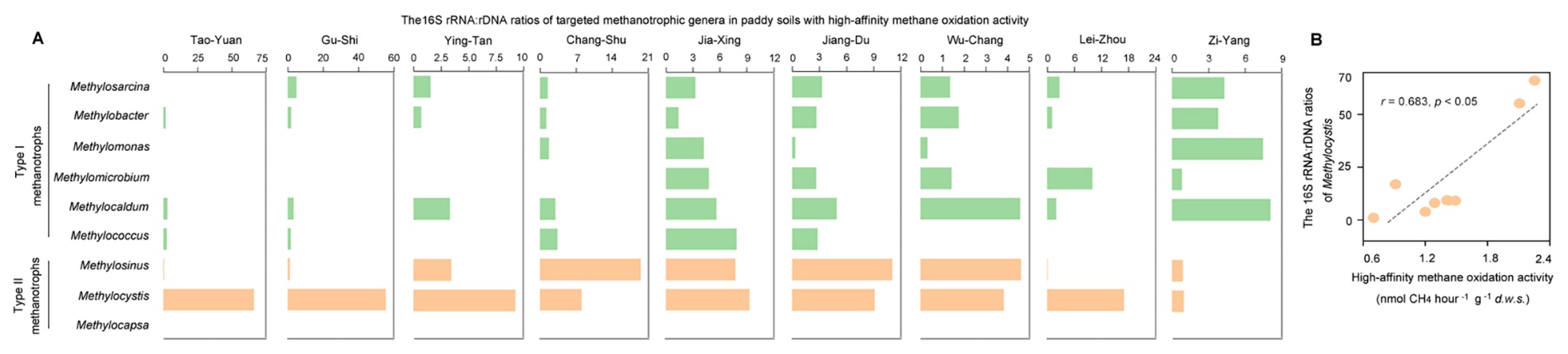


**Supplementary Figure 2 (A)** The ratios of 16S rRNA to 16S rDNA for methanotrophic genera in paddy soils with high-affinity methane oxidation activity. The 16S rRNA:rDNA ratios of methanotrophic genera are expressed as the ratios of the relative abundance of targeted methanotrophic 16S rRNA to the relative abundance of targeted methanotrophic 16S rDNA in each sample. **(B)** Correlation between high-affinity methane oxidation activity and the 16S rRNA:16S rDNA ratios of *Methylocystis* in the paddy soils (*n* = 9). The nonsignificant correlations between high-affinity methane oxidation activity and 16S rRNA:rDNA ratios of other methanotrophic genera (*p* > 0.05) are described in Supplementary Table 5. Statistical analysis was performed using Spearman’s rank-based correlation.


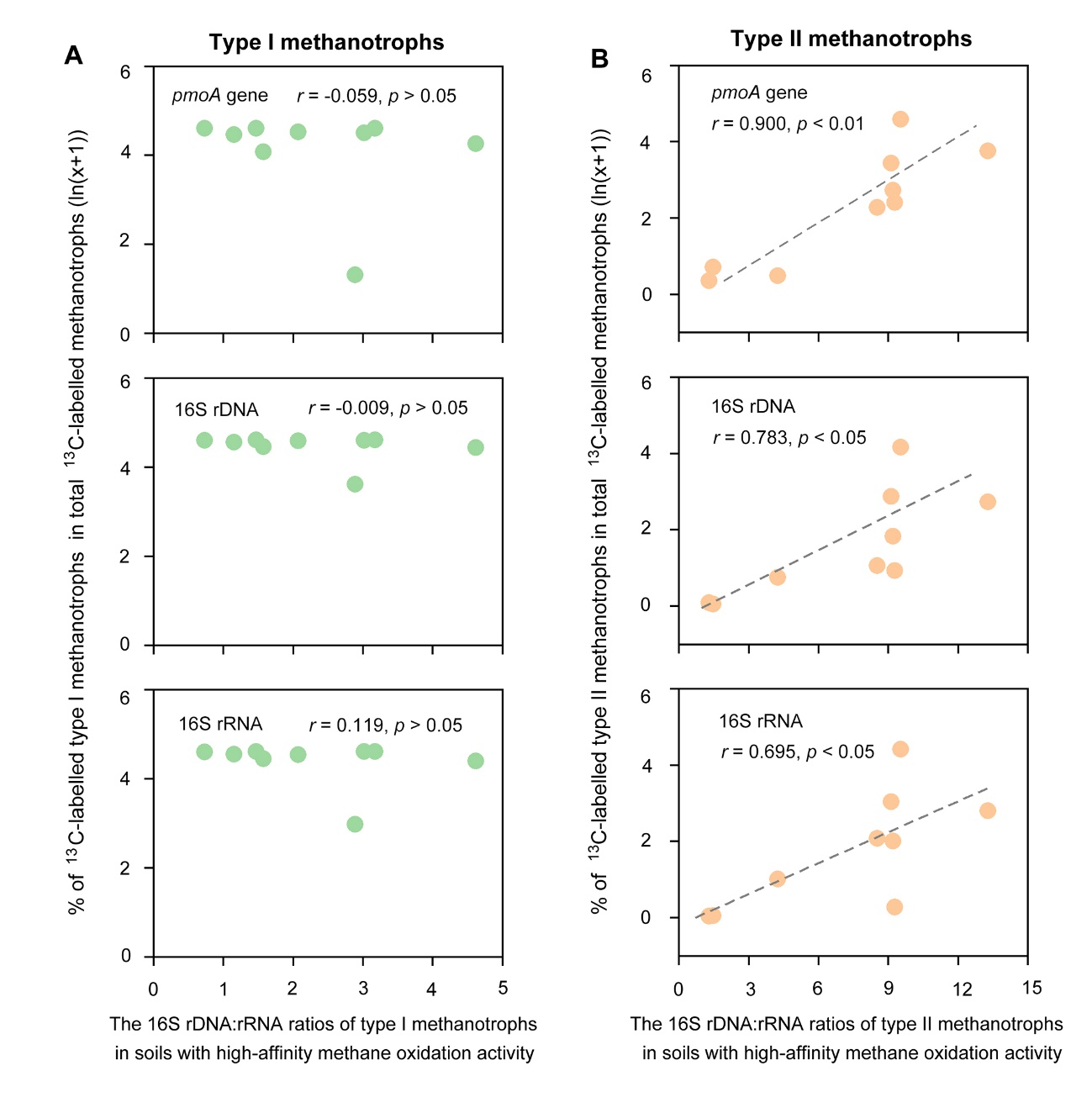


**Supplementary Figure 3 Correlations between the ratios of 16S rRNA:16S rDNA of methanotrophs and the relative abundance of ^13^C-labelled type I methanotrophs (A) and ^13^C-labelled type II methanotrophs (B) in paddy soils with high-affinity methane oxidation activity (*n* = 9).** The relative abundance of ^13^C-labelled methanotrophs was log-transformed before statistical testing. Statistical analysis was performed using Spearman’s rank-based correlation.


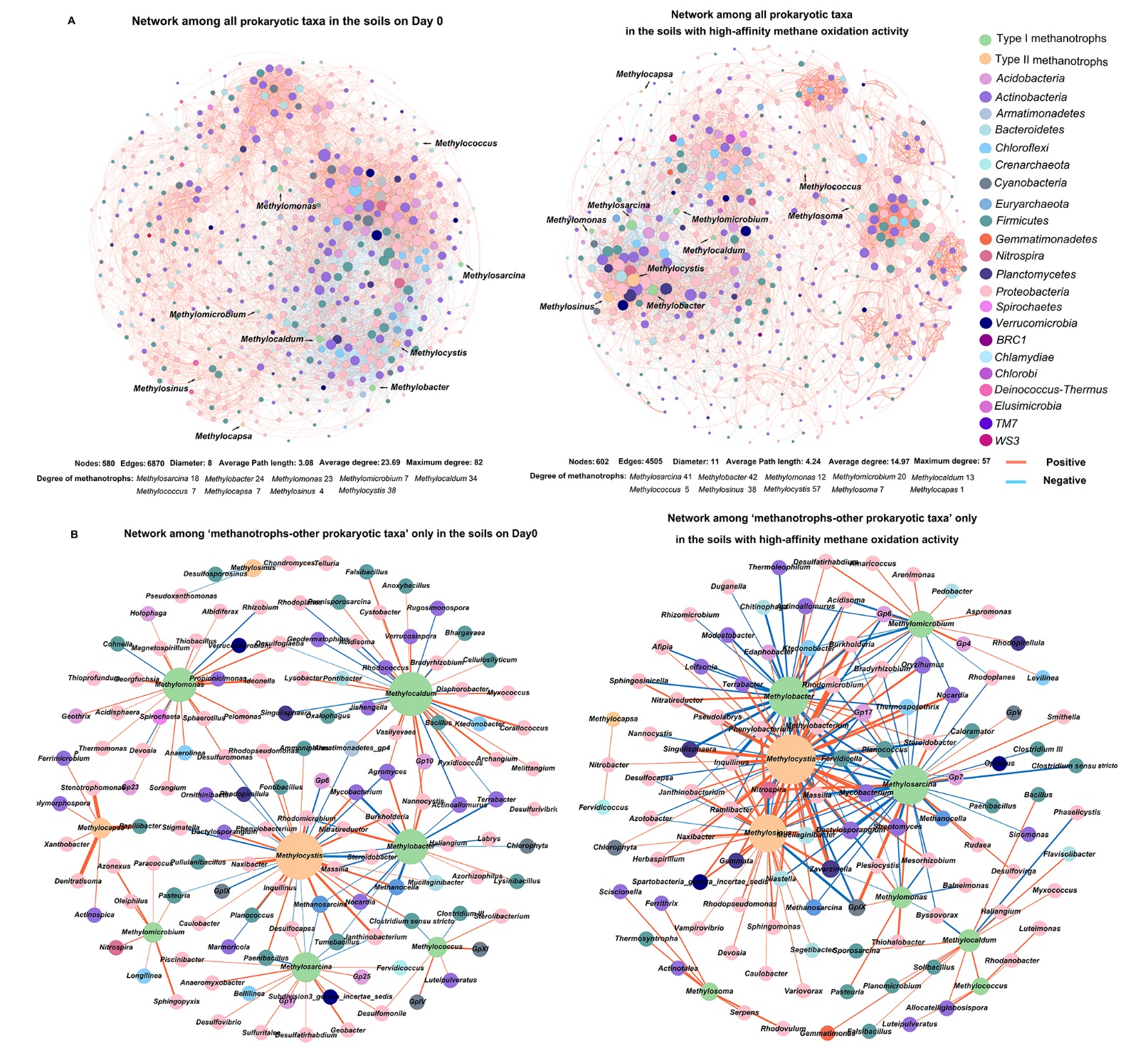


**Supplementary Figure 4 Co-occurrence network analysis showing the associations among all prokaryotic taxa (A) and among ‘methanotrophs-other prokaryotic taxa’ only (B) in the paddy soils.** The nodes represent the prokaryotic microorganisms at the genus level. Links between the nodes indicate significant correlations (Spearman’s correlation > 0.6, *p* < 0.05). The bigger the node, the higher the degree (the number of network links for the node). The networks among ‘methanotrophs-other prokaryotic taxa’ (B) were the subsets of the networks among all taxa (A). The networks among ‘methanotrophs-other prokaryotic taxa’ only (B) were generated using the taxa that were significantly correlated with methanotrophs. The network analysis is based on the relative abundance of all identified genera, expressed as the percentage of targeted genus-related 16S rRNA to the total 16S rRNA in paddy soils.

**Supplementary Table 1. Information of sampling sites**

| Sampling Sites | Coordinates | Rice harvests per year | Climate zone | Annual mean temperature (°C) | Maximum water-holding capacity  (g g^-1^) |  |
| --- | --- | --- | --- | --- | --- | --- |
| Tao-Yuan | 28°55′N, 111°27'E | 2 | subtropics | 17.5 | 0.65 |  |
| Gu-shi | 28°57′N, 111°31'E | 2 | subtropics | 17.5 | 0.72 |  |
| Ying-Tan | 28°13′N, 116°49'E | 2 | subtropics | 19.1 | 0.52 |  |
| Chang-Shu | 31°34′N, 120°43′E | 1 | subtropics | 17.7 | 0.60 |  |
| Jia-Xing | 30°38′N, 120°46'E | 1 | subtropics | 17.9 | 0.54 |  |
| Jiang-Du | 32°25′N, 119°42'E | 1 | subtropics | 16.8 | 0.55 |  |
| Wu-Chang | 44°59′N, 127°05'E | 1 | mid temperate | 3.0 | 0.51 |  |
| Lei-Zhou | 20°33′N, 110°04'E | 2 | tropics | 24.1 | 0.59 |  |
| Zi-Yang | 30°05′N, 104°34'E | 1 | subtropics | 18.1 | 0.64 |  |

**Supplementary Table 2. The quantity and purity of nucleic acids extracted from paddy soils**

| Sampling  Sites **a** | DNA | | |  | RNA | | |
| --- | --- | --- | --- | --- | --- | --- | --- |
|  | Concentrations  (μg g^-1^ *d.w.s.*) | A260/A280 **b** | A260/230 **c** |  | Concentrations  (μg g^-1^ *d.w.s.*) | A260/A280 | A260/230 |
| Tao-Yuan | 22.4 | 1.50 | 1.59 |  | 4.37 | 1.69 | 1.55 |
| Gu-shi | 19.9 | 1.90 | 1.35 |  | 8.43 | 1.74 | 1.59 |
| Ying-Tan | 9.31 | 1.75 | 1.44 |  | 4.17 | 1.84 | 1.40 |
| Chang-Shu | 14.6 | 1.84 | 1.52 |  | 6.62 | 1.61 | 1.59 |
| Jia-Xing | 8.38 | 1.94 | 1.39 |  | 4.65 | 1.63 | 1.62 |
| Jiang-Du | 14.7 | 1.92 | 1.32 |  | 5.40 | 1.87 | 1.49 |
| Wu-Chang | 13.8 | 1.90 | 1.29 |  | 2.30 | 1.62 | 1.35 |
| Lei-Zhou | 2.61 | 1.58 | 1.19 |  | 0.58 | 1.48 | 1.14 |
| Zi-Yang | 11.3 | 1.84 | 1.57 |  | 1.40 | 1.74 | 1.32 |

**a** The data represent the average values of nucleic acids from 10,000 ppmv-amended microcosms ( *n* = 6).

**b** Absorbance ratio between nucleic acids (260 nm) and both humic acids and proteins (280 nm).

**c** Absorbance ratio between nucleic acids (260 nm) and both humic acids and salts (230 nm).

**Supplementary Table 3. The estimated atmospheric methane consumption rate in paddy soils**

|  | High-affinity methane oxidation activity (nmol CH_4_ hour^-1^ g^-1^ *d.w.s.*) **a** | Estimated atmospheric methane consumption rate (kg CH_4_ ha^-1^ year^-1^) **b** |
| --- | --- | --- |
| Tao-Yuan | 0.225 | 6.034 |
| Gu-Shi | 0.211 | 5.650 |
| Ying-Tan | 0.143 | 3.824 |
| Chang-Shu | 0.129 | 3.455 |
| Jia-Xing | 0.141 | 3.770 |
| Jiang-Du | 0.149 | 3.987 |
| Wu-Chang | 0.120 | 3.212 |
| Lei-Zhou | 0.091 | 2.444 |
| Zi-Yang | 0.070 | 1.873 |

**a** The high-affinity methane oxidation activity was calculated based on the amount of atmospheric methane that can be oxidised in the first 3 h as shown in Figure 2.

**b** The soil covers an area of ~1.256 × 10^-3^ m^2^ with a depth of ~0.5 cm in the microcosms. It is assumed that the high-affinity methane oxidation activity of the 2 cm surface soils could be induced in the rice field (Cai et al., 2016). Estimated atmospheric methane consumption rate (kg CH_4_ ha^-1^ year^-1^) = High-affinity methane oxidation activity (nmol CH_4_ hour^-1^ g^-1^ *d.w.s.*) × 6 g *d.w.s.* × 10^-9^ × 16 g mol^-1^ × 10^-3^ / (1.256 × 10^-3^ m^2^) / 0.5 cm × 2 cm× 10^4^ m^2^ × 24 hours day^-1^ × 365 days

**Supplementary Table 4. High-throughput sequencing of *pmoA* genes in paddy soils**

Supplementary Table 4 is presented in the Excel named ‘Supplementary Tables’.

**Supplementary Table 5. High-throughput sequencing of 16S rRNA genes (16S rDNA) and 16S rRNA transcripts (16S rRNA) from the total DNA and the total RNA, respectively.**

Supplementary Table 5 is presented in the Excel named ‘Supplementary Tables’.

**Supplementary Table 6. High-affinity methane oxidation activity versus methanotrophic community relationships**

| Methanotrophic taxa | | High-affinity methane oxidation activity versus methanotrophic activity **a** | | | |  | High-affinity methane oxidation activity versus methanotrophic abundance relationships **d** |
| --- | --- | --- | --- | --- | --- | --- | --- |
|  |  | 16S rRNA:rDNA ratios **b** | ^13^C-labelled methanotrophs **c** | | |  |  |
|  |  |  | ^13^C-labelled *pmoA* gene | ^13^C-labelled 16S rDNA | ^13^C-labelled 16S rRNA |  |  |
| Total methanotrophs |  | 0.583 | / | / | / |  | -0.367 |
| Type I methanotrophs | Toal type I methanotrophs | 0.117 | -0.921** | -0.860** | -0.695* |  | -0.550 |
|  | *Methylosarcina* | 0.262 | -0.633 | -0.483 | -0.517 |  | -0.550 |
|  | *Methylobacter* | 0.017 | -0.300 | -0.218 | -0.100 |  | -0.200 |
|  | *Methylomonas* | -0.029 | -0.228 | -0.237 | -0.128 |  | 0.126 |
|  | *Methylomicrobium* | 0.143 | / | -0.350 | -0.128 |  | / |
|  | *Methylocaldum* | -0.250 | -0.531 | 0.500 | 0.283 |  | -0.317 |
|  | RPCs | / | 0.435 | / | / |  | 0.567 |
| Type II methanotrophs | Total type II methanotrophs | 0.967** | 0.933** | 0.850** | 0.778* |  | 0.750* |
|  | *Methylocystis* | 0.683* | 0.933** | 0.850** | 0.778* |  | 0.750* |
|  | *Methylosinus* | 0.083 | 0.733* | 0.834** | 0.849** |  | 0.633 |
|  | *Methylocapsa* | / | / | / | 0.548 |  | / |
|  | *pmoA2* | / | 0.548 | / | / |  | 0.827** |

**a** The methanotrophic activity was explored by examining 16S rRNA:rDNA ratios of methanotrophs and by documenting the relative abundance of ^13^C-labelled methanotrophs in paddy soils with high-affinity methane oxidation activity. Correlations between high-affinity methane oxidation activity and methanotrophic activity were estimated using Spearman's correlation analyses. The relative abundance of ^13^C-labelled methanotrophs was log-transformed before statistical testing. *, *p* < 0.05; **, *p* < 0.01.

**b** 16S rRNA:rDNA ratios of methanotrophs in the 10,000 p.p.m.v. methane-amended paddy soils with high-affinity methane oxidation activity.

**c** ‘^13^C-labelled *pmoA* gene’ represents the relative abundance of methanotrophs expressed as the percentage of the targeted methanotrophic taxa-related *pmoA* genes to total *pmoA* genes in the ^13^C-labelled DNA from the ‘heavy’ fraction of the ^13^CH_4_-labelled microcosms with high-affinity methane oxidation activity. '^13^C-labelled 16S rDNA' represents the relative abundance of targeted methanotrophs expressed as the percentage of the targeted methanotrophic taxa-related 16S rDNA to the total methanotrophic 16S rDNA in the ^13^C-labelled DNA from the ‘heavy’ fraction of the ^13^CH_4_-labelled microcosms with high-affinity methane oxidation activity. '^13^C-labelled 16S rRNA' represents the relative abundance of targeted methanotrophs expressed as the percentage of the targeted methanotrophic taxa-related 16S rRNA to the total methanotrophic 16S rRNA in the ^13^C-labelled RNA from the ‘heavy’ fraction of the ^13^CH_4_-labelled microcosms with high-affinity methane oxidation activity.

**d** Methanotrophic abundance represents the *pmoA* gene copies of targeted methanotrophic taxa expressed as the *pmoA* gene copies of total methanotrophs based on qPCR multiplied by the relative abundance of the targeted methanotrophic taxa-related *pmoA* genes based on high-throughput sequencing. The methanotrophic abundance was log-transformed before statistical testing. Correlations between high-affinity methane oxidation activity and methanotrophic abundance were estimated using Spearman's correlation analyses (*n* = 9). *, *p* < 0.05; **, *p* < 0.01.

**Supplementary Table 7. High-throughput sequencing of 16S rDNA in the fractionated DNA**

Supplementary Table 7 is presented in the Excel named ‘Supplementary Tables’.

**Supplementary Table 8. High-throughput sequencing of 16S rRNA in the fractionated RNA**

Supplementary Table 8 is presented in the Excel named ‘Supplementary Tables’.

**Reference**

Cai, Y., Zheng, Y., Bodelier, P.L.E., Conrad, R., Jia, Z., 2016. Conventional methanotrophs are responsible for atmospheric methane oxidation in paddy soils. Nature Communications 7, 11728.
